# COVID-19 Pandemic Response in a Migrant Farmworker Community: Excess Mortality, Testing Access and Contact Tracing in Immokalee, Florida

**DOI:** 10.5334/aogh.3859

**Published:** 2022-09-07

**Authors:** Neha Limaye, Brennan Ninesling, Frantzso Marcelin, Cody Nolan, Walter Sobba, Matthew Hing, Emily Ptaszek, Fernet Léandre, Daniel Palazuelos

**Affiliations:** 1 Department of Medicine, Brigham and Women’s Hospital, Boston, MA, USA; 2 Harvard T.H. Chan School of Public Health, Boston, MA, USA; 3 University of South Florida Morsani College of Medicine, Tampa, FL, USA; 4 Department of History, Steven J. Green School of International & Public Affairs, Florida International University, Miami, FL, USA; 5 New York University Grossman School of Medicine, New York, NY, USA; 6 David Geffen School of Medicine at UCLA, Los Angeles, CA, USA; 7 Partners In Health, Boston, MA, USA; 8 Peak Vista Community Health Centers, Colorado Springs, CO, USA; 9 Harvard Medical School, Boston, MA, USA; 10 Division of Global Heath Equity, Brigham and Women’s Hospital, Boston, MA, USA

**Keywords:** migrant health, migrant seasonal farmworkers, pandemic response, community health, language inequities

## Abstract

**Background:**

Migrant and seasonal farmworkers face enormous barriers to health and have been a particularly vulnerable population during the COVID-19 pandemic, but their pandemic experiences and potential inequities have not been well studied.

**Objectives:**

We aimed to assess the impact of COVID-19 in Immokalee, Florida, a community with a significant population of migrant and seasonal farmworkers. We evaluated for differences in pandemic experience by language, a known barrier to healthcare, to inform and strengthen future public health efforts.

**Methods:**

First, to estimate the burden of COVID in the area, we conducted a descriptive analysis of data on COVID-19 deaths for Collier County from May-August 2020. We then surveyed a cross-sectional, randomized representative sample of 318 adults living in Immokalee from March-November 2020 to assess socio-demographics, workplace conditions, sources of information, ability to follow guidelines, and experiences with testing and contact tracing programs. Results were compared across language groups.

**Findings:**

Average excess mortality in Collier County was 108%. The majority surveyed in Immokalee had socio-demographic factors associated with higher COVID risk. Non-English speakers had higher workplace risk due to less ability to work from home. Haitian Creole speakers were less likely to be tested, though all participants were willing to get symptomatic testing and quarantine. Those participants who tested positive or had COVID-19 exposures had low engagement with the contact tracing program, and Spanish-speakers reported lower quality of contact tracing than English speakers.

**Conclusions:**

The community of Immokalee, FL is a vulnerable population that suffered disproportionate deaths from COVID-19. This study reveals language inequities in COVID testing and contact tracing that should be targeted in future pandemic response in Immokalee and other migrant farmworker communities.

## 1. Introduction

### 1.1. Background

Migrant and seasonal farmworkers (MSFW) face enormous structural barriers to health, including poverty, food insecurity, poor working conditions, high occupational hazard, and limited access to healthcare [1]. Although data are lacking, those available suggest that MSFW experience inequities in multiple health outcomes [2,3,4]. At work they face challenging environmental conditions including heat, sun, pesticide exposure, and dust and particle inhalation [1]. Additionally, most MSFW are uninsured and must overcome language barriers, long work hours, and lack of transportation to attend health appointments, with care often only available at Federally Qualified Health Centers (FQHC) [5]. Consequently, the majority of MSFW do not have primary care physicians [6].

The COVID-19 pandemic has only deepened pre-existing health inequities. Data demonstrate disproportionate rates of COVID-19 morbidity and mortality among Black, Latinx and immigrant populations, who make up the majority of MSFW in the U.S [7,8,9]. MSFW are at especially high risk for COVID-19 exposure and subsequent infection due to high density housing and ‘essential worker’ status, which has exempted them from the protection of working from home enjoyed by other industries [10,11]. Typical working conditions are not conducive to distancing, as many MSFW work side-by-side and share transportation to and from work [12]. Once infected, spread within MSFW communities is facilitated by lack of testing and contact tracing, and by housing conditions which preclude distancing or effective quarantine [13]. Finally, those infected are at higher risk of severe COVID-19 disease due to underlying health conditions that predispose them to morbidity and mortality.

Florida houses one of the largest populations of MSFW in the country, and though the plight of MSFW during the COVID-19 pandemic has gained media attention, there is minimal data around the impact of the pandemic on MSFW communities. Specifically, though it is known that MSFW are a high-risk group, there is no data on the true death rates from COVID-19 for MSFW and there is little information regarding specific workplace conditions, sources of COVID-19 information, ability to follow guidelines, nor their experiences with testing and contact tracing. All of this information is key for improving public health programming.

### 1.2. Study Site: Immokalee, Florida

Immokalee is a rural community located in southwest Florida’s Collier County, the center of the nation’s tomato growing industry. The total population fluctuates with the agricultural seasons and is estimated at about 25,000, with 37.4% living below the poverty line [14]. Census data suggests 22% of the population works in agriculture and related fields, though other sources estimate as many as 15,000–20,000 MSFWs live in Immokalee for some parts of the year [14,15,16]. These discrepancies likely exist as MSFWs often live in temporary or mobile housing, which are underrepresented in census data [1]. Residents are primarily from Mexico, Central America, and Haiti; 68% of the population speak Spanish at home and 13% speak Haitian Creole, along with some bilingual or monolingual speakers of indigenous languages [6,17]. Previous studies in Immokalee have noted that residents face increased everyday stressors and workplace risks that can negatively impact health [18,19].

**Figure 1:**
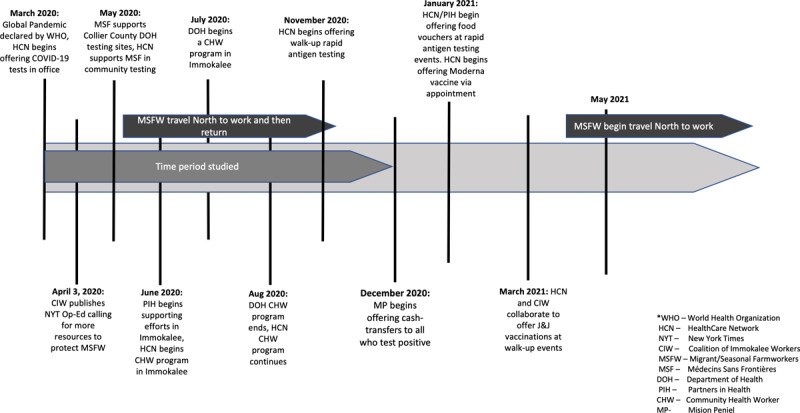
Timeline of COVID-19 Related Services in Collier County, Florida, March 2020–May 2021.

Occupational, economic, and linguistic factors confer substantial vulnerability to COVID-19 infection in Immokalee; despite its small size, Immokalee was the Florida zip code with the highest number of COVID-19 cases in the state in June 2020 [20]. Positivity rates at the time were as high as 36% compared to 5.6% in the state [21]. After observing the disease burden and difficulties in accessing COVID-19 testing and contract tracing among MSFWs, the Coalition of Immokalee Workers (CIW)—a local human rights organization—facilitated partnerships between the Collier County Department of Health (DOH), Partners in Health (PIH), Doctors Without Borders (Medicins Sans Frontieres (MSF)), and a local FQHC (Collier Health Services, Inc., d/b/a Healthcare Network (HCN)). The DOH led all contract tracing efforts in Immokalee, and offered nasal PCR testing by appointment until November 2020, with results given via an English-language online portal which asked for a social security number. Starting in November 2020, HCN began offering walk-up rapid testing in Immokalee, with results given within an hour by Spanish- and Haitian Creole-speaking staff. Both HCN and DOH created community health worker programs for COVID-19 outreach, but all positive results from HCN’s testing were forwarded to the DOH’s contact tracing system (see timeline in Figure 1 below).

In this study, we first aimed to estimate the impact of COVID-19 on Immokalee, FL by calculating excess mortality from publicly available data. Then, we conducted a household survey to collect sociodemographic information and assess community experiences with workplace conditions, access to testing, sources of information, and the DOH contact tracing program. Given the linguistic diversity in Immokalee and MSFW populations in general, and limited English proficiency as a known barrier to care, we evaluated for differences in experience by language.

## 2. Methods

### 2.1. Descriptive Analysis

We collated data from the Florida Department of Health and Medical Examiners Offices for Collier, Lee, Hendry, Glades and Orange Counties. First, we tabulated the total deaths from COVID-19 for residents of Collier County. Data for the Immokalee zip code alone were not available. We then compared deaths for Collier County during May-August 2015–2019 with deaths from May-August 2020. Excess mortality was calculated in accordance with CDC guidelines, where death data were grouped weekly to account for temporal effects [22]. Excess mortality was calculated for each week and then summed together to find excess mortality for the period beginning 4/27/20 and ending 8/16/20. Data were disaggregated by age (under 60 or ≥ 60 years) and sex.

### 2.2. Questionnaire

The questionnaire was created using demographic and social screening tools from the National Agricultural Workers Survey and various farmworker healthcare organizations [7,8,9]. Spanish- and Haitian Creole-speaking CIW staff reviewed the tool for understandability by the local population. The final questionnaire assessed the following thematic areas [dataset 23]:

- Demographic and socioeconomic information
- Sources of news and information on COVID-19
- Ability to follow COVID-19 precautions
- Experiences with contact tracing

### 2.3. Sample Size

Our target population was adults living in Immokalee during the months of March-November 2020. We estimated a population of 12,000 adults in Immokalee, a 95% confidence interval, a precision of 5%, and a prevalence of 15–20% of the population meeting requirements to be contacted by contact tracers (given an estimated local COVID-19 prevalence of 8%) to calculate a prevalence sample size. From our initial prevalence sample size of 193–241 participants, we accounted for intercorrelation that occurs when surveying multiple people per household. We used an intra-cluster correlation (ICC) of 0.1–0.33 and an estimated household size (m) of ~4 to calculate a design effect of 1.3–2, giving us a final sample size goal of 300–350 participants.

To choose addresses, we first obtained a publicly available address list from the Collier County Property Appraiser’s office. Then, given that many MSFW live in temporary or mobile housing [1] that is likely underrepresented in census data, we examined the public address list and added apartment complexes and clusters of mobile homes that were missing. These additional 669 addresses were added in consultation with local organizations, like CIW, who were familiar with the community. We then extracted 350 addresses from this compiled list using a random number generator. Study staff visited each address, and after exhausting that first address list, extracted 100 further addresses at a time to reach a sample size of 300–350 participants.

### 2.4. Study Procedures

From January 18-March 11, 2021, study staff (FM and BN) visited addresses from the random address list on weekends and evenings, when adults were most likely to be home from work. They wore appropriate personal protective equipment including a mask and eye protection at all times and conducting interviews outside to maintain social distance. They conducted multiple surveys per household as household members may have had different experiences, and it is common for multiple families in Immokalee to cohabitate.

Study staff started each visit with an initial screening to confirm each participant 1) was 18 years or older, 2) lived in Immokalee, and 3) lived in Immokalee for at least 2 weeks between March-November 2020. If inclusion criteria were met, study staff obtained verbal informed consent in the participants’ preferred language. FM is bilingual in Haitian Creole/English and BN is bilingual in Spanish/English. Verbal consent was obtained due to the population’s literacy level and to maintain participant anonymity. If participants preferred to participate at a different time or no adults were home, one follow-up visit was done at the same address. All households visited received a paper pamphlet detailing available health resources in their preferred language.

Questionnaires were administered on a secure tablet by the study staff. Participants’ responses were recorded by study staff directly into REDCap at the time of survey administration [24,25]. The data were checked weekly by the co-investigators to ensure internal validity. The study was reviewed and exempted by the Institutional Review Boards at Mass General Brigham (Protocol 2020P003045) and the Harvard School of Public Health (IRB20-1755). All data was de-identified and published online [dataset 23].

### 2.5. Data Analyses

All analyses were conducted utilizing R (Version 4.0.2, The R Foundation, 2021) and RStudio (Version 1.3.959, RStudio Team, 2021). Chi-square and Fisher’s exact tests were used to compare variables as appropriate. Of note, data from a question on sick leave was not analyzed due to participant misinterpretation of the question.

## 3. Results

### 3.1. Descriptive Analysis

As shown in Table 1, analysis of Collier County mortality data from April 27^th^ through August 16^th^ revealed an average excess mortality of 108% (167 excess deaths). When data were disaggregated by sex alone, age alone, and both sex and age, excess mortality findings were largely consistent (107%, 115%, and 115%, respectively).

**Table 1:** Results for total recorded deaths, predicted deaths, and estimated excess deaths in Collier County.


	MAY 2020					JUNE 2020				JULY 2020					AUGUST 2020		TOTAL
			
WEEK	1	2	3	4	5	6	7	8	9	10	11	12	13	14	15	16	

Date	4/27–5/3	5/4–5/10	5/11–5/17	5/18–5/24	5/25–5/31	6/1–6/7	6/8–6/14	6/15–5/21	6/22–6/28	6/29–7/5	7/6–7/12	7/13–7/19	7/20–7/26	7/27–8/2	8/3–8/9	8/10–8/16	

Total all-cause recorded deaths, no.	0	5	20	24	12	16	25	16	27	23	30	32	22	29	37	1	319

Predicted deaths, no.	0	2.4	9.6	4.5	13.5	15.3	13.0	14.5	14.2	11.2	8.1	12.1	11.0	13.5	10.7	0.4	154

Excess deaths, no.	0	2.6	10.4	19.5	0.0	0.7	12.0	1.5	12.8	11.8	21.9	19.9	11.0	15.5	26.3	0.6	167

Excess deaths,%	0	108	108	433	0	5	92	10	90	105	270	164	100	115	246	150	108


### 3.2. Study Population

**Figure 2:**
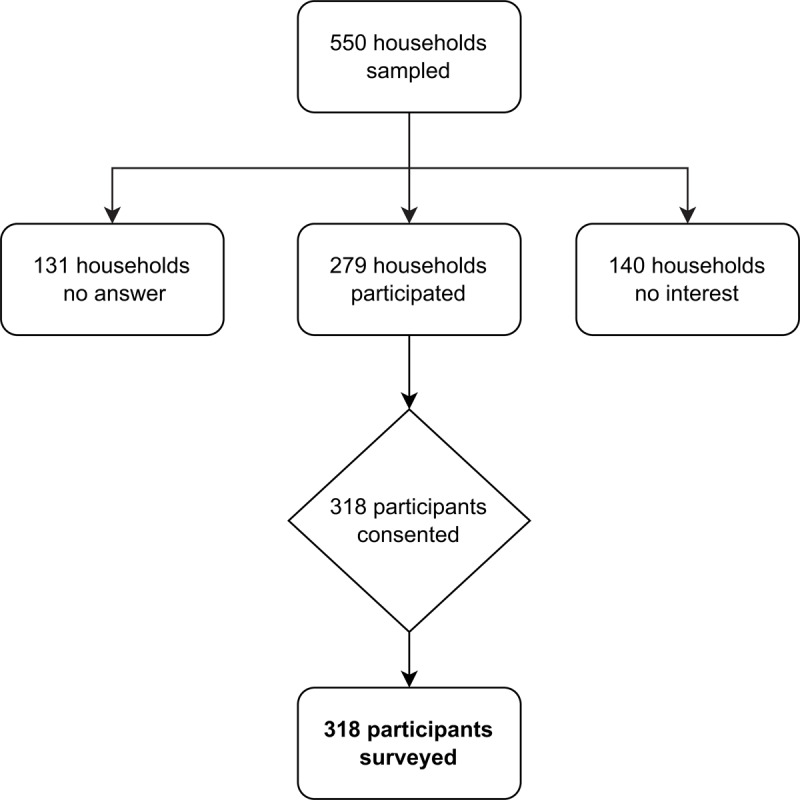
Flow Diagram of Participant Recruitment.

Of 550 households randomized, 131 (23.8%) did not answer the door, 140 (25.5%) were not interested in participating, and 279 (50.7%) agreed to participate. From these 279 households, 318 individuals consented to participate and were surveyed (Figure 2).

Baseline participant and household demographics are depicted in Table 2. All race/ethnicity/language data is per participants’ self-identification. Spanish (42.1%) was the most frequent preferred language, with English (37.3%) and Haitian Creole (18.7%) accounting for most other participants. Nearly one-fifth of participants reported food insecurity during the past month (19.2%). The mean household size was 3.95 persons with homes averaging 0.856 bedrooms per person and 0.483 bathrooms per person. Twenty-five percent of those surveyed were farm or packinghouse workers, 16.7% were unemployed, and many in the other category worked in service occupations including childcare, cleaning, maintenance, and sales.

**Table 2:** Demographic characteristics of survey participants.


CHARACTERISTIC	PARTICIPANTS	

	N*	%

**Gender**	N = 312	

Female	165	52.9%

Male	147	47.1%

**Age group (yrs)**	N = 313	

18–24	34	10.9%

25–34	76	24.3%

35–44	73	23.3%

45–64	96	30.7%

65–80	29	9.3%

81+	5	1.6%

**Race/Ethnicity**	N = 316	

White	10	3.2%

Black	76	24.1%

Latino/a	213	67.4%

Indigenous	1	0.3%

Bi- or Multi-racial	11	3.5%

Prefer not to answer	2	0.6%

Other	3	0.9%

**Preferred language**	N = 313	

English	118	37.7%

Spanish	133	42.5%

Haitian Creole	59	18.8%

Mam	3	1.0%

**Educational attainment**	N = 314	

No formal schooling	26	8.30%

Kindergarten-5th grade	42	13.40%

6–8th grade	44	14.00%

9–12th grade	161	51.30%

Beyond high school	41	13.10%

**Housing situation**	N = 317	

No housing	2	0.60%

Housed but housing insecure	83	26.20%

Housed	230	72.60%

Other	2	0.60%

**Housing characteristics (mean)**		

Household members	3.95	

Bathrooms	1.52	

Bathrooms per person	0.48	

Bedrooms	2.79	

Bedrooms per person	0.86	

**Food insecurity during last month**	N = 318	

Yes	61	19.20%

**Primary occupation**	N = 318	

Farmworker	64	20.10%

Other	64	20.10%

Construction	25	7.90%

Landscaping	18	5.70%

Packinghouse worker	16	5.00%

Healthcare worker	13	4.10%

Food service	13	4.10%

Maintenance or custodian	9	2.80%

Driver	8	2.50%

Painter	7	2.20%

Education	7	2.20%

Agricultural supervisor	4	1.30%

Casino Employee	3	0.90%

Unemployed	53	16.70%

Retired	14	4.40%


* N ranges between 312–318 based on lack of response to certain questions.

### 3.3. Essential Worker Status and Workplace Policies

Table 3 details participants’ occupational status and policies. Most employed individuals in all language groups were categorized as essential workers by the state of Florida based on their occupation [26]. Twenty-six percent of English speakers stated they were offered the option to work from home during the pandemic. By contrast, only 3% of both Spanish and Haitian Creole speakers reported being given the option to work from home (p < 0.001 English vs. Spanish; p = 0.0023 English vs. Haitian Creole).

**Table 3:** Self-reported workplace policies of participants and essential worker status, stratified by primary language spoken.


	ANSWER	PRIMARY LANGUAGE SPOKEN						COMPARISON	
	
		ENGLISH		SPANISH		HAITIAN CREOLE		ENGLISH/SPANISH (*p*)	ENGLISH/CREOLE (*p*)
		
		%	NO.	%	NO.	%	NO.		

**Essential Worker**	Yes	68.60%	81	76.70%	102	66.10%	39		

	No	31.40%	37	23.30%	31	33.90%	20	0.1522	0.7329

**Option to work from home?**	No	74.40%	67	97.00%	96	97.10%	34		

	Yes	25.60%	23	3.00%	3	2.90%	1	**<0.0000**	**0.0023**

**PPE at work?**	No	15.90%	14	26.50%	26	19.40%	7		

	Yes	84.10%	74	73.50%	72	80.60%	29	0.0784	0.6388


*Note*: Boldface indicates statistical significance (p < 0.05).

### 3.4. COVID-19 Testing Experiences

Table 4 shows participants’ testing experiences, compared by preferred language. Of English, Spanish, and Haitian Creole speakers, 38.1%, 48.1%, and 57.6% respectively reported that they had never been tested for COVID-19. Significantly fewer Haitian Creole than English speakers reported being tested (p = 0.014). English and Spanish speakers reported being tested at similar sites, primarily by the DOH, while Haitian Creole speakers were more likely to report being tested at the HCN Immokalee Clinic or HCN-affiliated mobile testing sites (p < .0001). English speakers were more likely to report being tested at sites outside of Immokalee, namely Fort Myers and Naples, compared to Spanish and Haitian Creole speakers.

**Table 4:** Participant experiences with COVID-19 testing, stratified by primary language spoken.


SURVEY QUESTION	ANSWER	PRIMARY LANGUAGE SPOKEN						COMPARISON	
	
		ENGLISH		SPANISH		HAITIAN CREOLE		ENGLISH/SPANISH (*p*)	ENGLISH/CREOLE (*p*)
		
		%	NO.	%	NO.	%	NO.		

Have you been tested for COVID-19?	Yes	61.90%	73	51.90%	69	42.40%	25		

	No	38.10%	45	48.10%	64	57.60%	34	0.1112	**0.0139^a^**

Where were you tested?	DOH	23.90%	21	25.70%	19	7.10%	2		

	Immokalee Clinic	18.20%	16	36.50%	27	60.70%	17		

	Naples	20.50%	18	16.20%	12	14.30%	4		

	Fort Myers	22.70%	20	10.80%	8	0.00%	0		

	Other	14.80%	13	10.80%	8	17.90%	5	**0.0547**	**<0.0000^b^**

How long for results?	<24 hours	31.50%	23	22.10%	15	56.00%	14		

	2–3 days	26.00%	19	25.00%	17	16.00%	4		

	4–6 days	11.00%	8	10.30%	7	12.00%	3		

	1 week+	31.50%	23	42.60%	29	16.00%	4	0.4967	0.1617^b^

What was the result?	Positive	27.40%	20	26.10%	18	0.00%	0		

	Negative	71.20%	52	73.90%	51	96.00%	24	0.8514	**0.0026^b^**

If you or another person in your household was showing symptoms such as fever, cough, fatigue, would you get tested for COVID-19?	Yes	87.30%	103	89.50%	119	91.40%	53		

	No	11.90%	14	3.00%	4	6.90%	4		

	Not sure	0.80%	1	7.50%	10	1.70%	1	**0.0132**	0.4292^b^

If you were to be unable to isolate in your current housing for COVID-19, would you be open to temporarily going to a supportive isolation shelter?	Yes	53.40%	63	65.40%	87	66.70%	38		

	No	38.10%	45	29.30%	39	24.60%	14		

	Not sure	8.50%	10	5.30%	7	8.80%	5	0.0885	0.0702^a^


*Note*: Boldface indicates statistical significance (p < 0.05). ^a^ Chi square. ^b^ Fisher’s exact.

There were no differences in the length of time that lapsed before results were available, though 31.5% of English and 42.6% of Spanish speakers reported waiting one week or more before receiving their results. More positive COVID-19 test results were reported for English-speaking participants (27.4%) than Haitian Creole-speaking participants (0%), though no significant difference was reported between English and Spanish speaking (26.1%) participants.

When asked whether participants would utilize testing resources if exposed to COVID-19, a large share of Spanish (89.5%), Haitian Creole (91.4%) and English speakers (87.3%) reported they would be willing to be tested. Most English (53.4%), Spanish (65.4%), and Haitian Creole speakers (66.7%) reported that they would be willing to isolate themselves in a temporary shelter if necessary and they were unable to do so inside their own home.

### 3.5. Quality of Contact Tracing

Per the DOH guidelines at the time of the study, those who tested positive for COVID-19 should have been called by the DOH to trace close contacts, inquire about their ability to quarantine, and provide information about local resources supporting quarantine. Calls were expected to be completed in the patient’s preferred language. Table 5 reflects participants’ experiences with the contact tracing process. No Haitian Creole speakers from our sample reported having tested positive. Only 35% of English speakers and 33% of Spanish speakers who tested positive were asked for names and phone numbers of individuals with whom they had been in close contact. Seventy percent of English speakers were asked about their ability to safely quarantine, compared to only 39% of Spanish speakers (p = 0.041). Forty-five percent of English speakers reported being provided information on local resources helping with quarantine, compared to 28% of Spanish speakers. Only 26% of English speakers reported being asked about their language preference compared to 82% of Spanish speakers, suggesting that most calls started in English and switched to Spanish if the recipient stated they did not understand English.

**Table 5:** Contact tracing experiences of participants, stratified by primary language spoken.


SURVEY QUESTION	ANSWER	PRIMARY LANGUAGE SPOKEN				COMPARISON
	
		ENGLISH		SPANISH		ENGLISH/SPANISH (*P*)
	
		%	NO.	%	NO.	

Participants who tested positive			20			18

Contact tracing occurred^a^	Yes	35.00%	7	33.30%	6	1

	No	60.00%	12	66.70%	12	

Quarantine guidance given^b^	Yes	70.00%	14	38.90%	7	**0.0409**

	No	20.00%	4	61.10%	11	

Connected to resources^c^	Yes	45.00%	9	27.80%	5	0.3133

	No	50.00%	10	72.20%	13	

Asked language preference	Yes	26.30%	5	81.80%	9	**0.0183**

	No	57.90%	11	18.20%	2	

Language of phone call	English	94.70%	18	9.10%	1	**<0.0000**

	Spanish	5.30%	1	90.90%	10	

Participants with positive close contact			42		24	

Informed of positive contact	Yes	33.30%	14	41.70%	10	0.6005

	No	64.30%	27	58.30%	14	


^a^ Did they ask for assistance in identifying the names and phone numbers of individuals you were in contact with? ^b^ Did they ask about your ability to safely isolate and quarantine in your current housing situation? ^c^ Did they provide information on any resources that exist to help assist with isolation or quarantine? *Note*: Boldface indicates statistical significance (p < 0.05).

Individuals that were identified as close contacts of someone that had tested positive for COVID-19 were also supposed to be called about their exposure and need to quarantine. Thirty-three percent of English speakers and 42% of Spanish speakers reported being called by the DOH about their positive close contact.

## 4. Discussion

In this first study of COVID-19 impact on a MSFW population, we found high excess mortality and high COVID-19 risk, with low testing and contact tracing rates and multiple language-based disparities despite many actions from a coalition of community organizations. The 108% excess mortality rate in Collier County calculated in this study is extremely high. In comparison, average excess mortality in Florida was estimated to be 15.5% from March to September, with a peak of 38.1% in August [27], while nationwide data showed an average of 18.5% excess deaths from March through the end of July [28]. While we cannot discern how many of these excess deaths were specifically from Immokalee, we know it is an especially vulnerable community within the county, as demonstrated by our data. With an average household size of ~4 people with shared bathrooms, over 20% with food and housing insecurity, and a preponderance of essential workers, the risk of COVID-19 infection was and continues to be high in this community [10]. For non-English speakers, that risk is even higher, as our data show they are less frequently able to work from home.

Confronted by this excess disease burden in a highly vulnerable population, the stakeholder groups came together rapidly and worked towards a COVID-19 response. Through their combined efforts, a new system for testing, quarantine and contact tracing was put into place. Our data show the successes and challenges of setting up such a system in Immokalee, with lessons relevant to MSFW populations across the U.S.

The initial testing system was based on appointments scheduled via an English-only online portal. While most surveyed residents indicated willingness to test and quarantine, the data show marked language disparities in testing, with significantly lower testing rates in Haitian Creole speakers. The HCN helped address this disparity a few months into testing by initiating rapid antigen testing at mobile field sites run by Haitian Creole- and Spanish-speaking staff. Our data show that this change may have facilitated a higher testing rate in the Haitian Creole population; amongst the number of Haitian Creole speakers tested, the majority received tests at these HCN rapid-test sites. However, the data show overall low testing rates for all language groups despite these collective efforts, demonstrating a need for even more accessible testing for predominantly MSFW populations.

Our data also highlight challenges with initiating a contact tracing program. Contact tracing is a key strategy for interrupting COVID-19 transmission and providing linkage to quarantine resources for vulnerable individuals [29,30], but the percentage of participants in this sample that received contact tracing calls was much lower than the 80% benchmark endorsed by recent studies [31]. Language disparities were also present in contact tracing: Spanish-speaking individuals were significantly less likely to be asked by contact tracers about their ability to safely isolate. We were unable to collect any data on Haitian Creole speakers’ experiences with contact tracing, because so few Haitian Creole-speaking respondents were tested. Without access to testing, the true burden of COVID-19 infections in the Haitian population is unknown, and uninterrupted transmission is likely to have occurred.

These disparities in testing and contact tracing highlight the challenges that the population and coalition of community organizations faced while responding to the high COVID-19 rates in Immokalee. The disparities demonstrate the need for all collaborators in the larger health system to connect more effectively with vulnerable communities like MSFW and other essential workers, especially Haitian Creole speakers who are known to be particularly marginalized.

To the best of our knowledge, this is the first study to examine community experiences with COVID-19 for MSFW in Florida and the first to evaluate a COVID-19 contact tracing program using probability sampling methods. The population sampled was representative of the diverse Immokalee community with study demographics that parallel 2019 Census Data regarding gender and race distribution and household size [32]. This study is well-powered to capture the contact tracing experiences of the population, especially as we anticipated more clustering than was present in our sample.

In terms of future public health responses both in Immokalee and other MSFW communities, it will be key to employ strategies that attend to differential access, vulnerability, and experiences. For example, several programs across the United States indicate promises of linking social support with contact tracing for vulnerable populations and represent an important target for intervention for future health emergencies [31,33]. The groups working in Immokalee are currently implementing various social support programs, and further research is necessary to understand the effects of this approach. Further research on the Haitian Creole population’s experience will also be essential in planning future responses that better reach the most vulnerable. Moving forward, rapid upscaling of testing access, quality improvement of contact tracing, and community vaccination are needed to prevent continued disproportionate COVID-19 spread and death in vulnerable MSFW populations as new variants of SARS-CoV-2 surge.

### 4.1 Limitations

First, though we added additional mobile homes and apartments to create a more representative sample, most addresses came from publicly available housing data, which likely missed some MSFW living in mobile and temporary housing. While our sample included many essential workers, only 20% were MSFW, which was less than anticipated. Also, the survey response rate was just over 50%, raising the possibility of non-response bias. Our suspicion is that non-respondents were more likely to view outreach efforts as intrusive during a pandemic and/or have concerns about migration status; we hypothesize that non-respondents were probably less likely to be reached by public health programs. Additionally, respondents who were migratory from June-October for farm work may have been less likely to receive services. Finally, three participants whose primary language was Mam (a Central American Indigenous language) completed the questionnaire in Spanish, potentially affecting their answers. Future surveys should include all languages in Immokalee. There may have been participants who would have preferred answering in Mam or another Indigenous language if offered this option, as there are known bilingual speakers in Immokalee [17].

## 5. Conclusion

Overall, this study quantifies the impact of COVID-19 on Immokalee and elucidates the individual, household, and occupational factors that place this community at especially high risk. The data show that despite coordinated efforts from a committed group of collaborators, significant language-based inequities impacted the risk of contracting COVID-19, testing rates, and receiving high-quality contact tracing. These inequities are a proxy for the disproportionate barriers faced by non-English speaking populations in Immokalee to access care.

## Additional File

The additional file for this article can be found as follows:

10.5334/aogh.3859.s1 Dataset. Questionnaire and dataset on dataverse.
